# Text-Book of Human Physiology

**Published:** 1876-07

**Authors:** 


A Text-Book of Human Physiology; Designed for the Use of Practitioners and Students of Medicine. By Austin Flint, Jr., M.D., Professor of Physiology and Physiological Anatomy in the Bellevue Hospital Medical College, New York; Fellow of the New York Academy of Medicine; Member of the Medical Society of the County of New York, etc., etc. Illustrated by three lithographic plates, and three hundred and thirteen wood cuts. D. Appleton & Co., 549 and 551 Broadway, New York, 1876, and for sale by Burke & Hancock, 26 Alabama St., Atlanta, Ga.

No work of recent date has given us more real pleasure and profit than the one before us. The large five volume treatise of Professor Flint, upon the Physiology of Man, is here admirably condensed in a single volume of 978 pages. thus making this great work accessible to every practitioner and student of medicine. It is, in fact, the cream gathered from the five volumes of the Physiology of Man, without loss or detriment to the subject matter. The bibliographical citations and matters of historical interest in the large work are expunged from this, and subjects elaborately discussed in the former are here concisely and carefully condensed.

The work will be eagerly sought by practitioners and students of medicine, as an indispensable accession to their list of text-books.

The *newness* of the work, both in the subject-matter and the illustrations, is truly refreshing. We notice illustrations from the original works of Fabricus, Harvey and Asellius. Among them is an elaborate illustration of the valves of the veins, copied and reduced from a figure in the original work of Fabricus, published in 1687. We also notice beautiful microscopical photographs from the United States Army Medical Museum. The work is beautifully and elegantly illustrated and adds greatly to its interest and value. We are especially pleased to see that Prof. Flint has avoided the mistake of very many authors in minutely and laboriously elaborating their own peculiar views and discoveries. Certainly none more than himself has a just right to be proud, if not vain, of his contributions to the science of physiology. No more prominence is given the author's original and valuable observations upon "The Location and Source of Want of Air in the General System, the New Excretory Function of the Liver, the Function of Glycogenesis, the Influence of Muscular Exercise upon the Elimination of Urea," etc.

Among the 313 illustrations in the work, there are many new and novel to the general reader, and add greatly to its interest and merits. The work will become deservedly popular as a book of reference to the general practitioner, and a textbook for the student of medicine.

